# Using context to improve protein domain identification

**DOI:** 10.1186/1471-2105-12-90

**Published:** 2011-03-31

**Authors:** Alejandro Ochoa, Manuel Llinás, Mona Singh

**Affiliations:** 1 Department of Molecular Biology, Princeton University, Princeton, USA; 2 Department of Computer Science, Princeton University, Princeton, USA; 3 Lewis-Sigler Institute for Integrative Genomics, Princeton University, Princeton, USA

## Abstract

**Background:**

Identifying domains in protein sequences is an important step in protein structural and functional annotation. Existing domain recognition methods typically evaluate each domain prediction independently of the rest. However, the majority of proteins are multidomain, and pairwise domain co-occurrences are highly specific and non-transitive.

**Results:**

Here, we demonstrate how to exploit domain co-occurrence to boost weak domain predictions that appear in previously observed combinations, while penalizing higher confidence domains if such combinations have never been observed. Our framework, Domain Prediction Using Context (dPUC), incorporates pairwise "context" scores between domains, along with traditional domain scores and thresholds, and improves domain prediction across a variety of organisms from bacteria to protozoa and metazoa. Among the genomes we tested, dPUC is most successful at improving predictions for the poorly-annotated malaria parasite *Plasmodium falciparum*, for which over 38% of the genome is currently unannotated. Our approach enables high-confidence annotations in this organism and the identification of orthologs to many core machinery proteins conserved in all eukaryotes, including those involved in ribosomal assembly and other RNA processing events, which surprisingly had not been previously known.

**Conclusions:**

Overall, our results demonstrate that this new context-based approach will provide significant improvements in domain and function prediction, especially for poorly understood genomes for which the need for additional annotations is greatest. Source code for the algorithm is available under a GPL open source license at http://compbio.cs.princeton.edu/dpuc/. Pre-computed results for our test organisms and a web server are also available at that location.

## Background

Protein domains are fundamental units of protein structure, function, and evolution. As a result, domain prediction is an important first step in the annotation of protein sequences [1]. Enhancements in domain identification improve protein annotations, as domains are often associated with specific cellular functions, and novel domain predictions can either newly predict or further refine functional predictions [2,3]. Furthermore, some domains are known to be associated with structures and thus their identification can be used for inferring protein structure [4,5]. Domain predictions are also the starting point for a range of more sophisticated analyses, including comparative genomics of domain families in diverse organisms [6-8], studies of the evolution of protein and domain structure and function [9-11], prediction of protein-protein interactions [12-15] and identification of complex evolutionary relationships [16].

The majority of proteins contain more than one domain [17]. Domains occur in different combinations, and the domain composition of multidomain proteins is critical for their specialized functions. Domains do not form random combinations, and indeed a limited fraction of domain pairs and triplets are highly recurrent [18]. While the mechanisms that lead to new domain combinations have been extensively explored and the analysis of observed domain combinations has received significant recent attention (reviewed in [19]), this information has not yet been widely used for domain prediction.

Here, we exploit the tendency of certain domains to co-occur with each other in order to improve domain identification. While domains within any given sequence are typically identified by considering each domain family individually, domain co-occurrence or "context" is useful in detecting weak sequence similarity [20]. In particular, two domain families that frequently co-occur provide "positive context", and for a given sequence, if these domains are identified with low confidence individually, their weak signal can be amplified. Similarly, domain family pairs that have never been observed provide "negative context" and their occurrences can be penalized (but not necessarily eliminated), thereby preventing unnatural combinations of low scoring predictions and limiting false predictions.

We have developed a novel graph-theoretic framework that combines individual domain scores with pairwise scores derived from domain co-occurrence statistics, in order to find a set of domains that maximize an overall score. Our approach, dPUC (**D**omain **P**rediction **U**sing **C**ontext), uses Pfam [21] profile hidden Markov models (HMMs) [22] to score domains individually, along with a novel log-odds scoring system that captures the propensity of pairs of domains to be found in the same sequence. While we have developed our approach using Pfam, alternate libraries of domain profiles (e.g., SMART [23], Superfamily [4], or CDD [5]), as well as different pairwise context scoring schemes, can be readily incorporated.

We test dPUC via rigorous benchmarks on eight organisms, ranging from bacteria (Escherichia coli and Mycobacterium tuberculosis), to protozoa (yeast and Plasmodium species) and metazoa (human, fly, worm). We present the first large-scale demonstration that incorporating domain context improves domain predictions in organisms across the evolutionary spectrum. Overall, dPUC gains up to 11% more domains at noise rates comparable to the Standard Pfam's, and outperforms the recently-published method CODD (Co-Occurent Domain Discovery) which also incorporates domain context [24]. Further analysis demonstrates that dPUC's performance improvements are due in part to penalization of negative context as well as allowing context between repeated domains. Importantly, we also find that dPUC does not require much additional time beyond that necessary for Pfam to initially identify domains.

We have found that dPUC is particularly effective at improving domain predictions for the genome of the poorly annotated malaria parasite *Plasmodium falciparum*. For this parasite, we perform further testing and show that dPUC's predictions are consistent among orthologs in closely related *Plasmodium *species. Moreover, we have used dPUC to annotate *Plasmodium *proteins and have newly identified proteins taking part in core processes such as ribosomal assembly and other RNA processing events. Overall, our findings in *Plasmodium *and other genomes suggest that domain identification can be significantly improved by incorporating context, particularly for organisms with poorly understood genomes.

## Methods

### Data

#### Pfam database

Pfam 23 was downloaded from the website (http://pfam.sanger.ac.uk/). A list of nesting families was extracted from Pfam-A.seed, defining a set of "allowed overlaps" consisting of domains with overlapping amino acid ranges within a protein sequence. The domain architecture of a protein is defined as its ordered list of domains, including repeats. We parsed the Pfam-A.full file to obtain the complete domain assignments to Uniprot 12.5 sequences, and thereby the domain architectures used to compute domain context scores (described below).

#### Proteomes

We used the proteomes of several model organisms for our testing, along with those of several parasites of medical interest. The proteomes of *E. coli, M. tuberculosis, Saccharomyces cerevisiae, Caenorhabditis elegans, Drosophila melanogaster*, and *Homo sapiens*, were downloaded from Uniprot [25] 15.8 (15.10 for *M. tuberculosis*), by obtaining all sequences with the organism taxon numbers 83333, 1773, 4932, 6239, 7227, and 9606, respectively, the keyword "complete proteome" (keyword 181), and not including isoforms. The proteomes of the *Plasmodium *species *P. falciparum *[26], and *P. vivax *[27] were downloaded from PlasmoDB 6.0 [28], and sequences containing internal stop codons were removed. Consistency of predictions on the *Plasmodium *species were further tested by analysis on *P. knowlesi *[29], *P. chabaudi, P. berghei*, and *P. yoelii*, which were also downloaded from PlasmoDB.

### Approach

#### The dPUC model

For a given protein sequence, let *P *be a set of candidate domains (which we obtain by setting a permissive threshold on the HMMER domain E-values). For each domain *i *in *P*, let *H*_*i *_be the domain score of *i*, let *T*_*i *_be the domain score threshold for the family of domain *i*, and for each pair of domains *i *and *j*, let *C*_*ij *_be the context score between *i *and *j *(see dPUC context scores below). Let *D *⊆ *P *be a subset of domains, and then for each domain *i *we define its score with respect to this set of domains as

**Figure 1:**
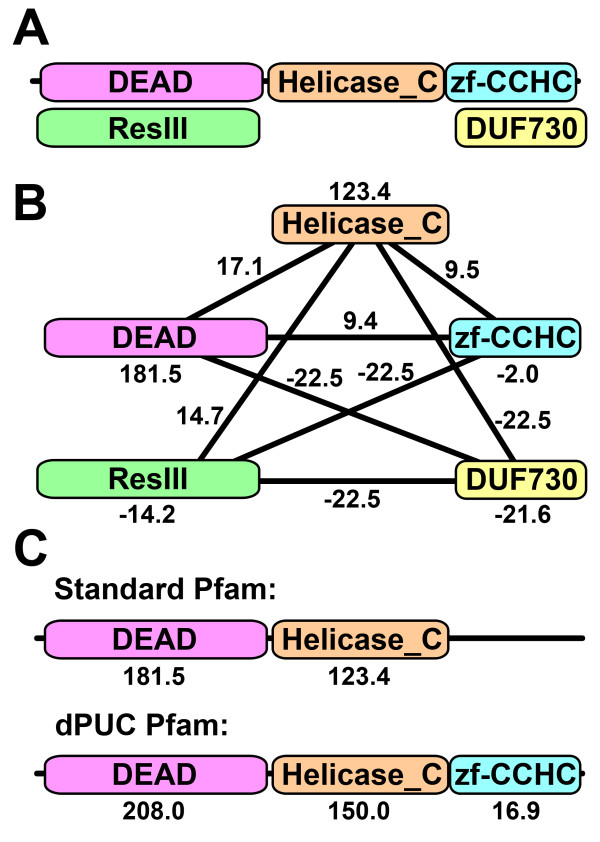
**Illustration of the dPUC framework using Pfam to identify initial domains**. **A**. We gather candidate domain predictions using Pfam with a permissive threshold. Domains are arranged in the x-axis by their amino acid coordinates, but the y-axis arrangement is arbitrary (there may be overlapping initial predictions). **B**. We build a network between candidate domains. Node weights are the normalized Pfam HMM scores of the corresponding domains (raw score minus the domain threshold). Edge weights between non-overlapping domains are set to our context scores. **C**. The Standard Pfam will make limited predictions, while dPUC may boost weak domains over the thresholds if they are in the correct context. The dPUC solution maximizes the sum of the node and edge weights, without overlaps, and each node must satisfy the Pfam thresholds. The final normalized domain scores are shown for each framework.

Our goal is to find the subset of domains *D *⊆ *P *that maximizes the "total score" *Σ*_*i *__∈ __*D *_*S*_*i,D*_, constrained so that *D *only contains "allowed overlaps" and each domain *i *in D satisfies the domain threshold *S*_*i,D *_*≥ 0*. Note that without context scores (that is, *C*_*ij *_*= 0 *∀*i,j*), the last inequality is reduced to the standard definition of a domain threshold. Our framework can be illustrated from a graph-theoretic point of view (Figure 1).

#### Formulation using ILP

We solve our combinatorial optimization problem with integer linear programming (ILP). In particular, we define a 0/1 variable *x*_*i *_for each domain *i*, and a 0/1 variable *x*_*ij *_for each pair domains *i *and *j*. If variable *x*_*i *_is set to 1, this indicates that domain *i *is included in our final set of predictions. Similarly, if variable *x*_*ij *_is set to 1, this indicates that the pair of domains *i *and *j *are in our final set of predictions. The score of each domain with respect to the other chosen domains is given by

Our goal is to maximize

where *T*_F_* is a Pfam-specific sequence threshold (see Additional File 1 "dPUC implementation details"). Though a theoretically difficult problem, we solve this ILP via a two pronged approach based on domain elimination and using lp_solve 5.5.0.14 [30]. See Additional File 1 Supplementary Methods for further information on solving the ILP and relevant details about the Pfam curated and domain-specific "gathering" thresholds, including the treatment of combined local and glocal domains.

#### dPUC context scores

We derive pairwise log-odds context scores using Pfam architectures found in Uniprot. These architectures are filtered to remove those that occurred in only one sequence, since they are more likely to be erroneous. Let *e*_*ip *_be the number of domains of family *i *in protein *p*, and let *e*_*p *_be the total number of domains in protein *p*. We obtain "normalized pair counts" *c*_*ij *_as follows:

where the sum over proteins *p *goes only over multidomain proteins, as single domain proteins would have zero denominators. Note that the normalization given above divides the pairs from each protein by the total number of domains of that protein (minus one), to compensate for inflated counts due to proteins with many domains. While there are many possible normalization schemes, our scheme keeps domains from counting themselves, and each protein *p *contributes *e*_*p *_counts to all *c*_*ij *_in total. These counts are turned into probabilities by setting

where *c = Σ*_*ij *_*c*_*ij*_, *α = 1 *is the regularization parameter (to handle the zero count case), and *n *is the number of domain families observed in the architectures (*≤ 10,340 *families in Pfam 23 due to architecture filtering). By construction, *Σ*_*ij *_*p*_*ij *_*= 1*. The null model probabilities are

since random domains appear approximately uniformly using *E*-value thresholds. The context scores are

with *b = 2*, to match the HMMER2 bit scores. From these equations we can derive that unobserved pairs (pairs with *c*_*ij *_*= 0*) are always penalized, since they are assigned the same large negative score of *-log*_*b*_*[1 + c/α]*. Finally, observed pairs with negative scores are instead set to zero. This way, observed pairs are never penalized. For Pfam 23, only 5 out of the 15,929 observed pairs had negative scores that were subsequently set to zero. We also experimented with other log-odds scoring schemes (see Additional File 1 Supplementary Results); however, most schemes did not significantly change the performance of the overall approach.

#### Empirical analysis of dPUC runtimes

Since each protein sequence is a separate problem, the problem can be parallelized over proteins. We considered the 25,047 proteins of *E. coli*, *M. tuberculosis*, *S. cerevisiae*, and *P. falciparum*, and ran each problem on a 2.66 GHz Intel processor with 8 GB RAM. Runtime is measured in wall clock time. The Standard Pfam (the bulk of which is HMMER processing) runs in 96.4 ± 41.4 seconds per protein (mean ± standard deviation), while the dPUC overhead (that is, not including HMMER) runs at an additional 0.0229 ± 0.865 s/protein. However, the runtime distributions have very long tails, as can be seen in Additional File 1 Figure S1. Nevertheless, we find that in 95% of the cases, the dPUC optimal solution is obtained within 0.015 s, and in 99.94% of the cases, the optimal solution is found within 1 s.

### Other approaches

#### Baseline methods

We tested three non-context methods that serve as natural baselines for dPUC. The first is the "Standard Pfam", which uses their curated and domain-specific "gathering" (GA) thresholds (details in Additional File 1 "Pfam relevant details"). Since the Standard Pfam produces a single data point, we created the "Pfam Extended GA" method, in which the GA thresholds are shifted by constant amounts (details in Additional File 1 "Pfam Extended GA thresholds"), allowing us to explore a range of noise cutoffs. Lastly, the "Pfam *E*-value" method uses domain *E*-value thresholds instead of the GA thresholds.

#### CODD context method

To contrast with dPUC, we implemented two simple context approaches that filter candidate domains. The first is based on CODD, which incorporates positive context information whereby a low scoring domain can be predicted based on co-occurrence with a higher scoring domain [24]. Given a network of positive context domain pairs, a set *G *of Pfam predictions that pass the gathering thresholds with no disallowed overlaps, and a set of candidate predictions *D*, this filter finds the set of final predictions *P *as follows. First we initialize *P = G*. For each domain *d *in *D *sorted ascending by *E*-value, we transfer *d *to *P *if *d *has positive context with any domain in *G *and *d *does not have disallowed overlaps with any domains in *P*. The second approach is a novel double positive and negative filter of our creation that we call nCODD, and is described in the Additional File 1 Supplementary Methods.

### Testing

We developed two approaches for assessing the performance of dPUC.

#### Estimated FDR

**Figure 2:**
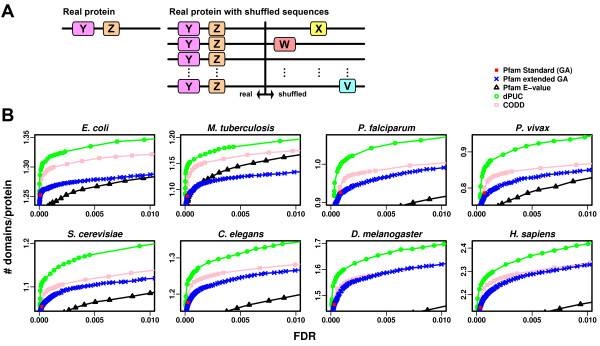
**dPUC predicts more domains over a range of FDRs**. **A**. Illustration of the FDR estimation procedure. For each original protein sequence, we make predictions on it and on twenty shuffled sequences concatenated to the original sequence, to allow "real" domains (Y, Z) to boost false predictions on the shuffled sequence (domains V, W, X) when using context. The estimated FDR is the ratio of false predictions per protein to the total number of predictions per protein. In this illustration, FDR ≈ (3/20)/(2) = 7.5%. **B**. The y-axis is the number of predicted domains per protein ("signal"), while the x-axis is the FDR ("noise"), so better performing methods have higher curves (more signal for a given noise threshold). dPUC (green circles) outperforms all non-context Pfam variations tested and the context method CODD.

In our first test, we compare the number of predictions from different methods (i.e., Standard Pfam, dPUC, and CODD) on real and shuffled protein sequences. The key idea is that domain predictions on shuffled sequences arise by chance alone, whereas predictions on real sequences give us the total number of predictions (true or false), and their ratio approximates the false discovery rate. We shuffle the residues of each protein separately, thereby preserving the amino acid composition of each sequence as well as the length distribution over each proteome. For the context methods (dPUC, CODD, nCODD), we run them on the real sequence concatenated to its shuffled sequence, but only count the number of predicted domains on the shuffled portion of the sequence. Therefore, we count the random predictions that might have been rewarded by positive context not only with other random predictions, but also with potentially real domains from the real sequence (Figure 2A). The shuffling is performed 20 times for each sequence.

We estimate the false discovery rate (FDR) as follows. Let *A *be the number of predictions per shuffled protein, and let *R *be the number of predictions per real protein. Then *FDR = A/R*, which is a common approximation of the FDR [31]. To calculate the FDR of the new dPUC domains only, let *A*_*n *_and *R*_*n *_be the quantities defined above when we use negative context only, and *A *and *R *when we use the *E*-value threshold of interest for candidate domains. The difference of the data corresponds almost entirely to the novel domains, ignoring the effect of the initial negative context elimination:

This benchmark is only appropriate for methods in which the orientation of the domains is unimportant, as it is for the methods tested here. We note that the Markov model of Coin *et al. *[20] is sensitive to the orientation of domains, so its performance cannot be measured by this benchmark; we also note that an implementation of this program is not available online. See Additional File 1 "Estimated FDR details" for further details on estimating the FDR.

#### Ortholog coherence scores

In our second test, we measure how often domains are predicted across orthologs. The key assumption is that real domains are very likely to be present in orthologs, whereas false domains are very unlikely to be. Therefore, the average "ortholog coherence" score is inversely related to the amount of spurious predictions. We chose the *Plasmodium *species' proteins because their sequence divergence is large enough to discard "coherent" false predictions due to high sequence similarity, yet the sequences are similar enough for orthologs to be identified easily, and domain architectures are largely conserved. Moreover, as we show below, our approach performs well in improving domain identifications in these species.

**Figure 3:**
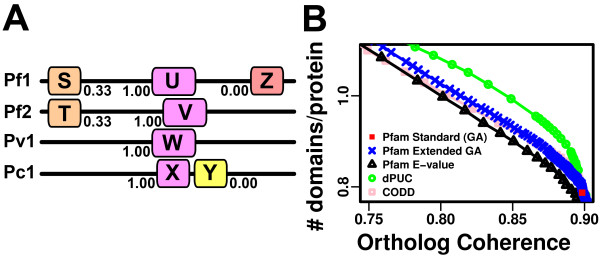
**dPUC predicts more domains over a range of Ortholog Coherence scores on Plasmodium species**. **A**. Illustration of scores. Domain predictions are made on hypothetical aligned orthologs and in-paralogs (Pf1, Pf2, Pv1, and Pc1). Color denotes domain family. Domain S overlaps T of the same family, so their scores are 1/3 (since they lack predictions in Pv1 and Pc1). In contrast, U is predicted 100% in its orthologs and in-paralogs. Y overlaps V but is not of the same family, so its score is zero. Similarly, Z does not overlap any domains. The score of this method is the average domain score on all proteins, ~0.58, while the average number of domains per protein is 2. **B**. The y-axis is the number of predicted domains per protein ("signal"), while the x-axis is the ortholog coherence score (inversely related with "noise"), so better performing methods have higher curves (more signal for a given noise threshold). dPUC (green circles) outperforms the other methods. Symbols and colors are as in **Figure 2**.

We computed the orthologous groups of six *Plasmodium *species using OrthoMCL 1.4 [32]. We obtained 5582 orthologous groups (32,250 proteins). We eliminated orthologous groups with more than 13 proteins to avoid constructing large alignments and to ignore the well-studied large paralogous families that are characteristic of *Plasmodium *species (including PfEMP1, RIFIN, STEVOR, in *P. falciparum *and VIR, YIR, KIR, and CIR in the other species, which may bias our results). This left us with 5523 groups with a total of 30,065 proteins. Each group was aligned with T-Coffee 8.14 [33], using the M-Coffee special mode which combines the alignments of T-Coffee, ProbCons 1.12 [34], and Muscle 3.6 [35]. The score of a domain is the fraction of times we observed overlapping domains (after mapping to the alignment) of identical family in the orthologs. The score of a method is the average domain score over all proteins (Figure 3A).

## Results

We chose eight diverse and representative organisms to test our method, including human, four model organisms, *D. melanogaster, C. elegans, S. cerevisiae*, *E. coli*, and several pathogens including the eukaryotic human malaria parasites *P. falciparum *and *P. vivax*, and the prokaryotic parasite *M. tuberculosis*. We test our method separately on each of these organisms since they have different and sometimes extreme biases in protein lengths, amino acid compositions, amino acid coverage by domains, and domain family content (Additional File 1 Figure S2).

### dPUC improves Pfam predictions across all tested organisms and across a range of FDRs

For a given method (e.g., Standard Pfam, dPUC, or CODD), we estimate its FDR as the ratio of the number of predictions made on shuffled sequences (when concatenated to real sequences, in the case of context methods), to the number of predictions made on the real sequences only. This concatenation approach allows noise in the shuffled sequence to be boosted by potentially real domains via context (Figure 2A). We test dPUC by varying the HMMER *E*-value threshold on its candidate domains. dPUC consistently enhances the performance of Standard Pfam across organisms and over the entire range of FDRs tested (Figure 2B). The Standard Pfam produces a single data point, and notably, its FDR is non-zero for all organisms. To explore Pfam's tradeoff between coverage and false positives, we vary the threshold to the HMMER *E*-value; this leads to fewer predictions on real sequences for the same FDR, showing that curation of Pfam thresholds has added value. We also vary the Pfam "gathering" thresholds by shifting them uniformly for all domain families, which performs better than *E*-value thresholds in all organisms except in *M. tuberculosis*, suggesting the Pfam curated domain thresholds are less appropriate for this diverged and compositionally biased (GC-rich) organism than they are for model organisms. Note that even when limiting the dPUC candidate domains to those predicted by the Standard Pfam (effectively when setting *E ≤ 0.001*, the leftmost datapoint of the dPUC curves), we see a sharp decrease in dPUC's FDR relative to the Standard Pfam; this improved performance can be directly attributed to the removal of false predictions using negative context scores.

The FDR as described above applies to the entire set of predictions. For dPUC with low HMMER *E*-value thresholds on the candidate domains, the FDR is the net effect of removing domains from the Standard Pfam through negative context, as well as adding new domains with positive context. Since negative context alone reduces the FDR of the predictions, the FDR of the new domains must be larger than the FDR of the whole. For dPUC with *E ≤ 1*, the FDR of the new domains only (see Methods) varies between 0.8-2%, depending on the organism (see Additional File 1 Table S1).

### dPUC outperforms simple filters incorporating context

We implemented an alternative context method, using filters, in which context scores are not defined. The first filter emulates the method CODD [24], in which candidate domains pass if they co-occurred with the domains that pass the Pfam gathering thresholds. Interestingly, the performance of CODD is similar to non-context methods in some organisms, and especially at high HMMER *E*-values thresholds (Figure 2B). We tested CODD using the published CODD positive context network, which notably lacks context between domains of the same family, and additionally removes observed domain pairs that do not occur more often than expected from the hypergeometric distribution. We note, however, that using the CODD filter with the dPUC network of positive context domain pairs improves the predictions compared to using this more limited CODD network (Additional File 1 Figure S3). Our previous analysis suggested that a negative filter (to mirror our negative scores) was necessary to enhance the FDR, and indeed, our approach of a double positive and negative filter, nCODD (see Additional File 1 Supplementary Methods), has better performance than CODD (Additional File 1 Figure S3). Nevertheless, nCODD falls behind dPUC in all organisms tested, with the exception of *D. melanogaster *and *H. sapiens*, in which performance is similar to dPUC's; this could be due to the overall better annotations of these two model organisms. In *P. falciparum*, nCODD is too aggressive and removes predictions from the real sequences as well as from the shuffled sequences, shifting the entire curve downward relative to dPUC. These results suggest that there is added value in our setup of scores and thresholds, compared to these simpler filter formulations.

### Performance of chosen context scores

Our context scores have a specific form (log-odds, see Methods) in which a pair score is logarithmically proportional to how often the domain family pair is observed in Standard Pfam. Although these log-odds scores seem naturally compatible with the HMMER log-odds scores, it is not certain that this is the form the scores should have. To test the importance of these scores, we shuffled the positive scores (three times), which resulted in decreased performance compared to our original context scores, but better performance than that of non-context methods (data not shown). Therefore, carefully choosing values for our scores is important, and our form, which weighs the evidence of co-occurrence per domain family pair, performs better than random scores from the same distribution. Additional score parameter variations are discussed in Additional File 1 "dPUC Pfam parameter robustness".

### dPUC increases Pfam coverage

We choose a threshold of *E ≤ 1 *to identify the candidate domains, which corresponds to an overall FDR of 0.03-0.2% across organisms; that is, this is within an order of magnitude of the Standard Pfam FDR of 0.01-0.09% (Additional File 1 Table S1). We then calculate the dPUC net percent improvements compared to the Standard Pfam (Table 1). The number of domains increases by 4-11%, with a trend roughly inverse to the level of initial coverage in each organism (Additional File 1 Figure S2D). Unique domain families increase by 3-6%, while repeated families increase at the higher rates of 12-40%. Amino acid coverage improves by 2-8% relative to the amino acids covered by the Standard Pfam. However, most new predictions appear in proteins that have Standard Pfam domains, since there are smaller increases in protein coverage (0.1-1.8%). Overall, the two *Plasmodium *species attain the highest increases in coverage, but even the best-annotated model organism, *E. coli*, experiences increases in coverage under all metrics (Table 1).

**Table 1:** dPUC increases domain predictions and amino acid coverage

	*E. c.*	*M. t.*	*P. f.*	*P. v.*	*S. c.*	*C. e.*	*D. m.*	*H. s.*
Domains	4.30	6.00	10.30	11.46	6.21	8.07	9.08	7.15
Domains unique families	2.62	2.91	5.73	5.60	3.38	3.66	4.38	3.43
Domains repeated families	26.56	39.61	23.83	37.48	23.79	19.95	18.26	12.34
Amino acids	2.38	4.13	7.25	7.66	3.14	4.74	5.63	3.63
Proteins	0.16	0.08	1.80	1.31	0.38	0.70	0.59	0.56

Percent increases of dPUC predictions relative to the Standard Pfam are given for each organism (*E. c*., *E. coli*; *M. t.*, *M. tuberculosis*; *P. f.*, *P. falciparum*; *P. v*., *P. vivax*; *S. c*., *S. cerevisiae*; *C. e*., *C. elegans*; *D. m*., *D. melanogaster*; *H. s*., *H. sapiens*) when considering all domains, first appearance of domains in a protein, subsequent occurrences of domains in a protein, amino acids covered by a domain, and all proteins with domain predictions. (See text.)

### dPUC leads to additional functional annotations

We used the MultiPfam2GO procedure [3] to obtain GO annotations from our domain predictions. This program uses a probabilistic approach to determine how sets of domains imply GO terms. We ran this procedure on the Standard Pfam and dPUC predictions, and filtered the results so that only the most specific GO terms remained (by removing all ancestors using all GO relationships).

In total, 2.5-7.8% of proteins had new or modified GO terms, depending on the organism. As we observed before with Pfam coverage, the two *Plasmodium *species attained the largest increases in GO terms. We present the summary of our data in Table 2 (detailed counts are in Additional File 1 Table S2). The vast majority of the original GO terms from Standard Pfam (over 97%) are preserved by dPUC with *E ≤ 1*. Additionally, we obtain a 1.2-4.0% increase in GO terms that are completely new, and a further 0.4-0.9% in GO terms that are more specific than previously existing GO terms. Our procedure also results in a negligible 0.2-0.7% of GO terms in dPUC becoming less specific than their Standard Pfam counterparts, and 0.3-1.4% GO terms being deleted. Similarly, most proteins with GO terms from Standard Pfam are unchanged by dPUC, but 1.8-5.0% of proteins have new or more specific GO terms, while only 0.3-1.3% of proteins have fewer or less specific GO terms. Finally, 0.3-1.5% of proteins experience both increased and decreased specificity of GO terms.

**Table 2:** dPUC predictions lead to novel or more specific Gene Ontology terms on proteins

	*E. c.*	*M. t.*	*P. f.*	*P. v.*	*S. c.*	*C. e.*	*D. m.*	*H. s.*
Same	98.51	98.27	96.86	96.01	98.28	97.07	96.26	96.68
New or more specific	1.80	1.98	5.05	5.00	2.04	3.07	3.40	3.13
Deleted or less specific	0.39	0.52	0.88	1.25	0.32	0.90	1.12	0.86
Mixed	0.28	0.49	1.07	1.53	0.72	1.17	1.61	1.36

Comparison of dPUC-based GO predictions with those based on the Standard Pfam. Values are percents relative to the number of proteins with GO terms in the Standard Pfam per organism. Each category is mutually exclusive, with "Mixed" specifying that both "new or more specific" and "deleted or less specific" GO terms occurred in the same proteins.

As expected, inspection reveals that most GO terms that are deleted or become less specific are a consequence of either domain replacement brought upon by positive context (which are usually accompanied by new GO terms) or domain removal due to negative context. In either case, we expect the new domain predictions to be more accurate, and the resulting removal of GO terms is welcomed. Interestingly, the addition of domains can also lead to GO term loss, which is a consequence of the MultiPfam2GO probabilistic model combined with incomplete training data (a detailed example is presented in Additional File 1 "Novel domain predictions may lead to GO term deletions with MultiPfam2GO").

### Domain coherence is enhanced across *Plasmodium* orthologs

We chose to focus on the *Plasmodium *parasites, the causative agents of malaria, due to their wide impact on human health, and also because our method showed the largest improvements in these organisms. A simple test of prediction quality is to ask if domains are predicted in orthologous sequences as well, since orthology information is not exploited by our method. Importantly, ortholog co-prediction is expected to be low for false predictions and high for real domains, providing us with an alternative measure of noise that does not depend on statistical simulations.

We defined an "ortholog coherence" score between 0 and 1, namely, the average fraction of times a domain is predicted in orthologous proteins (Figure 3A). We looked at the proteins of six *Plasmodium *species (*P. falciparum*, *P. vivax*, *P. knowlesi*, *P. chabaudi*, *P. berghei*, and *P. yoelii*) with orthologs or in-paralogs in these organisms as predicted by OrthoMCL, and plotted these scores against their number of domain predictions per protein for the same methods tested earlier (Figure 3B, Additional File 1 Figure S4). These coherence scores may be artificially low due to artefacts in the alignments or gene models (that is, exons might be missing in some orthologs), but on average all methods should be affected equally. Using dPUC with E ≤ 1 increases domain predictions by 11% at practically the same ortholog coherence (a 0.87% decrease) as the Standard Pfam. Coherence scores recapitulate our conclusions derived independently from our FDR analysis, displaying an increase in domain predictions over a wide range of ortholog coherence thresholds.

### Novel *P. falciparum *annotations

Careful manual analysis of novel domain predictions on the *P. falciparum *proteome led to the reannotation of 55 proteins, either due to novel Pfam domains that had not been predicted before (Table 3) or due to novel Pfam domains confirmed by other domain databases (Table 4). Our discoveries include the identification of orthologs to many core machinery proteins conserved in all eukaryotes, including those involved in ribosomal assembly and other RNA processing events, which surprisingly had not been previously known (full details in the Additional File 1 "New annotations on *P. falciparum*"). Three predictions find direct support in the literature. PF11_0086, the predicted "poly(A)-binding protein-interacting protein 1" PAIP1, has a strong yeast 2-hybrid interaction with PFL1170w [36], the annotated "poly(A)-binding protein" PABP1 homolog in *P. falciparum*. Additionally, the two DEAD-box helicases PFE1390w and MAL8P1.19, predicted to be the orthologs of ABSTRAKT and DBP10 respectively, agree with the assignments of a recently published curated list of *P. falciparum *helicases [37]. Such new functional annotations can serve as starting points for developing new therapeutic intervention strategies. Our suggestions have been submitted to PlasmoDB [28] as community annotations.

**Table 3:** Completely novel P. falciparum dPUC predictions lead to refined protein annotations

Protein ID	Standard Pfam domains	Additional dPUC domains	Current annotation (PlasmoDB 6.0)	Suggested reannotation (this study)
PFL0980w	CwfJ_C_1	CwfJ_C_2	conserved *Plasmodium *protein, unknown function	Debranching enzyme-associated ribonuclease (DRN1 ortholog), putative
PF13_0222	Metallophos	DBR1	phosphatase, putative	RNA lariat debranching enzyme (DBR1 ortholog), putative
PF11_0086	MIF4G	PAM2	MIF4G domain containing protein	Poly(A)-binding protein-interacting protein 1 (PAIP1 ortholog), putative
PFE1390w	DEAD, Helicase_C	zf-CCHC	RNA helicase-1	Post-translational mRNA regulation (ABSTRAKT ortholog), putative
PF08_0130	WD40	Utp13	WD-repeat protein, putative	U3 ribonucleoprotein component (PWP2 ortholog), putative
PF14_0456	WD40	Utp12	conserved *Plasmodium *protein, unknown function	U3 ribonucleoprotein component (DIP2 ortholog), putative
PF10_0128	WD40	Utp13	WD-repeat protein, putative	U3 ribonucleoprotein component (UTP13 ortholog), putative
PFI1025w	RRM_1	Lsm_interact	RNA binding protein, putative	U4/U6 snRNA-associated-splicing factor (PRP24 ortholog), putative
PFL0985c	DUF367	RLI	conserved protein, unknown function	Ribosome biogenesis regulator (TSR3 ortholog), putative
MAL8P1.19	DEAD, Helicase_C	DBP10CT	RNA helicase, putative	Ribosomal biogenesis RNA helicase protein (DBP10 ortholog), putative
PFE0560c	MORN	Avl9	MORN repeat protein, putative	Atypical Golgi transport protein (AVL9 ortholog) with MORN domains, putative
PFL1455w	DUF202, SPX	VTC	conserved *Plasmodium *protein, unknown function	Vacuolar transporter chaperone (VTC2/3/4 ortholog), putative
PFL2255w	TPR_2	F-box	conserved *Plasmodium *protein, unknown function	DNA replication origin binding protein (DIA2 ortholog), putative
PFF1070c	UPF0004, Radical_SAM	TRAM	radical SAM protein, putative	Ribosome or tRNA methylthiotransferase (RIMO or MIAB ortholog) or CDK5 regulatory subunit-associated protein 1, putative
PFL1045w	DUF814	FbpA	conserved protein, unknown function	FbpA domain protein, putative
MAL13P1.182	RanBPM_CRA	LisH	conserved *Plasmodium *protein, unknown function	GID8 ortholog, putative
MAL13P1.79		zf-CCCH, WD40	conserved *Plasmodium *protein, unknown function	CCCH zinc finger protein, putative
MAL13P1.37		zf-B_box	conserved *Plasmodium *protein, unknown function	Tripartite motif protein, putative

These dPUC domain predictions were novel relative to the Standard Pfam, SMART, and Superfamily, and coherent predictions were present in OrthoMCL orthologs or in-paralogs. dPUC predictions always contained the Standard Pfam domains, so only the additional domain predictions are listed. The number of repeats per family is not shown.

**Table 4:** Additional P. falciparum dPUC predictions lead to refined protein annotations

Protein ID	Standard Pfam domains	Additional dPUC domains	Suggested reannotation (this study)
PFE1240w	Radical_SAM, Wyosine_form	Flavodoxin_1	Wybutosine synthesis protein (TYW1 ortholog), putative
PFF1490w	THF_DHG_CYH_C	THF_DHG_CYH	Tetrahydrofolate dehydrogenase/cyclohydrolase (MTD1 ortholog, MIS1/ADE3 homolog without FTHFS domain), putative
MAL8P1.139	DDA1*	WD40	Regulator of (H+)-ATPase in Vacuolar membrane (RAV1 ortholog), putative
PF08_0124	CactinC_cactus	Cactin_mid	CACTIN homolog, putative
PF10_0152		NTP_transf_2, PAP_assoc	Non-canonical cytoplasmic specific poly(A) RNA polymerase protein (CID13 ortholog), putative
MAL13P1.170	NTP_transf_2	PAP_assoc	Non-canonical poly(A) RNA polymerase protein (PAP2/TRF5 ortholog), putative
PFI1560c	DUF21	CBS, cNMP_binding	Required for mitochondrial morphology (MAM3 ortholog), putative
PF10_0126		WD40	Phosphoinositide binding protein (HSV2/ATG18 ortholog), putative
PFI0510c	BRCT	IMS	DNA repair protein (REV1 ortholog), putative
MAL13P1.54	WD40	LisH	Alternative splicing regulator (SMU-1 ortholog), putative
PF14_0052	cobW	CobW_C	COBW domain-containing protein 1 (CBWD1 ortholog), putative
PF08_0012	SET, Pre-SET	YDG_SRA	Histone lysine N-methyltransferase, putative
PFE1445c		FG-GAP	T-cell immunomodulatory protein (human TIP homolog), putative
PFL0975w	IQ	RCC1	Unconventional myosin fused to IQ and RCC1 domains, putative
PF11_0276	Abhydro_lipase	Abhydrolase_1	Steryl ester hydrolase (TGL1/YEH1/YEH2 ortholog), putative
PF13_0190	Aha1_N	TPR_2, TPR_1	Chaperone binding protein, putative
PF11_0287	CRAL_TRIO	CRAL_TRIO_N	CRAL/TRIO protein, putative
PF11_0197	Ank	ACBP	Acyl-CoA-binding protein, putative
PF14_0647	TLD	TBC	Rab GTPase activator, putative
PFL0575w	Amino_oxidase, Thi4*	PHD	PHD finger and flavin containing amine oxidoreductase, putative
MAL13P1.246	E1-E2_ATPase	Cation_ATPase_C	E1-E2 ATPase, putative
PF11_0116		Nol1_Nop2_Fmu	Nol1/Nop2/Fmu-like protein, putative
MAL7P1.127		Pkinase	Rab GTPase activator and protein kinase, putative
PFC0425w		zf-C3HC4, PHD	PHD finger protein, putative
PFI0975c		RCC1	Regulator of chromosome condensation, putative
PFD0900w		RCC1	Regulator of chromosome condensation, putative
MAL7P1.132		Pkinase	Protein kinase, putative
PFF0810c		Ras	Ras GTPase, putative
PFL1990c		zf-CCHC, RRM_1	RNA binding protein, putative
PF07_0066		RRM_1	RNA binding protein, putative
PF13_0147		RRM_1	RNA binding protein, putative
PFF1120c		EGF	EGF-like membrane protein, putative
PF14_0262	WD40	TPR_1	WD40 and TPR repeats protein, putative
PFI0275w		WD40	WD40 repeat and EF hand protein, putative
PF10_0285		WD40	WD40 repeat protein, putative
PF11_0195		WD40	WD40 repeat protein, putative
PF14_0640		WD40	WD40 repeat protein, putative
MAL13P1.308		Arm	ARM repeat protein, putative

These dPUC predictions were novel compared to the Standard Pfam, and were consistent with existing domain predictions from SMART or Superfamily (and often present in orthologs too). The number of repeats per family is not shown.

dPUC predictions always contained the Standard Pfam domains, so only the additional domains are listed, *except *when marked with an asterisk (*; MAL8P1.139 has DDA1 in Standard Pfam but not in dPUC Pfam; PFL0575w has a Thi4 in Standard Pfam but it is replaced by another Amino_oxidase domain [belonging to the same Pfam clan] in dPUC Pfam).

All proteins have the current PlasmoDB 6.0 annotation of "conserved *Plasmodium *protein, unknown function" except: MAL8P1.139, PFI1560c, MAL13P1.246, MAL7P1.127 "conserved *Plasmodium *membrane protein, unknown function"; PFE1240w, PF11_0287 "conserved protein, unknown function"; MAL13P1.170 "nucleotidyltransferase, putative"; PF08_0012 "SET domain protein, putative"; PFF1120c "conserved Apicomplexan protein, unknown function"; PF14_0262 "probable protein, unknown function".

## Discussion

We have shown that domain identification can be greatly improved across a diverse set of organisms by exploiting domain co-occurrence information. Our method is more successful at increasing domains in genomes with lower domain coverage (Table 1 and Additional File 1 Figure S2D) and shows greatest improvements in the least annotated of the organisms we tested, the malaria parasites.

To our knowledge, there has been limited prior work examining the utility and systematic use of domain context for domain prediction [20], and to date such approaches have not been widely adopted, nor are they publicly available. Coin *et al. *developed a Markov model framework to score the dependence between domains in a sequence. However, their work presents two versions of the Markov model, each of which has opposing limitations: the first-order Markov model has few parameters, but its predictive power is limited; alternatively, their *k*^th^-order Markov model has more power (*k *= 5 gives twice as many predictions as *k *= 1), at the cost of an exponential (in *k*) increase in parameters, which become difficult to estimate accurately and increase the computational requirements of the approach. In contrast, dPUC allows interactions between all domains, as opposed to just the previous *k *domains, while keeping the parameter space small, simplifying parameter estimation and reducing our program's memory usage. Furthermore, the source code accompanying our work should facilitate further improvements in context-based identification of domains.

Recently, a method was published (CODD) that uses a list of "favored" domain pairs to predict lower-scoring domains in *P. falciparum *if high scoring domains that preferentially appear with them are found in the same sequence [24]. We have shown that dPUC significantly outperforms such a filter (Figure 2 and Figure 3), as it predicts many more domains at any fixed FDR. There are several important methodological contributions of dPUC that together explain this performance enhancement. First, our method penalizes (but does not necessarily eliminate) domain pairs that have never been observed before, whereas CODD uses only information about favored domain pairs. Second, our method uses domain family pair-specific log-odds scores and thresholds, while CODD treats all favored domain family pairs equally. Third, our method allows weak domains to boost each other in the absence of strong domains without any special treatment, while CODD has to be run separately to allow candidate domains to predict each other, and with different *E*-value thresholds since this is much more prone to false predictions. Fourth, our problem is combinatorial in nature, allowing domains with low *E*-values to overcome higher *E*-value candidates if the context is more favorable, while CODD simply prioritizes candidate domains by *E*-value. Fifth, CODD uses a limited positive context network that does not include repeating domains and eliminates pairs that do not have small enough p-values derived from the hypergeometric distribution. Although our benchmarks show that negative context and our more complete dPUC positive context networks are critical differences (Additional File 1 Figure S3 and Figure S4), these features alone do not explain dPUC's higher performance, suggesting that the other differences also play an important role. The importance of negative context is consistent with the fact that domain context is usually not transitive. In particular, we find that if a Pfam domain A co-occurs with domains B in at least one sequence in Uniprot and it co-occurs with domain C in at least one sequence, then domains B and C co-occur in at least one sequence only 15.7% of the time; however, domain pairs that do not co-occur can readily be predicted by methods that only reward favored domain pairs.

Other works, such as AIDAN [38] and a similar work [39], have also used domain context to refine domain predictions in a manner complementary to the work discussed here. In particular, while our method learns parameters from domain databases and uses these to make predictions on each protein sequence independently, AIDAN clusters sequences based on the original domain predictions, and refines these predictions by performing domain architecture alignments and evaluating sequence comparisons within these clusters when domains are missing or are mismatched. Further, the thresholds used by AIDAN are tuned so less than 0.05% of the new domain assignments are errors. The AIDAN approach can be used as a second step in any domain prediction pipeline, including our own, to further refine predictions by directly taking the sequence similarity of related proteins into account, and this is a promising avenue for future work.

Domain context is independent of the information that HMM scores capture, since an HMM score only uses the sequence the domain encompasses, ignoring the surrounding sequence that contains the rest of the domains, while domain context allows these other domains to affect each other. These two information sources do not overlap. In other words, HMMs capture so-called "vertical" information [40] while domain context captures "horizontal" information. This is similar to advances in remote homology detection due to the incorporation of secondary structure predictions in addition to HMM/profile information [40-42]. Fuzzy HMMs [43] have been proposed to model positional dependence within a domain, but currently these predictions are less tractable compared to classical HMM algorithms. We believe that incorporating horizontal information in HMM analysis will become more common, as it is evidently an important means for boosting subtle sequence signals.

We have implemented a log-odds system to score the co-occurrence of domain pairs. Indeed, log-odds scores are widespread in bioinformatics, and our scoring system is inspired by the log-odds scores used in local sequence alignment (LSA) [44]. We borrowed the concept that optimal scores ought to be log ratios of the "target" frequencies versus the "background" frequencies. Broadly speaking, there are many similarities in both problems, including that they score pairs and that the searches are both "local" (LSA seeks the best subsequences, while dPUC seeks the best subset of domains). However, an important assumption of the theory behind LSA is that different amino acid pairs are uncorrelated, whereas domain pair scores in dPUC are correlated, since solutions must be connected as a full clique (see the solution in Figure 1B). Our framework does not handle these correlations, and while we have demonstrated empirically that log-odds scores perform remarkably well, we expect that a better theoretical foundation for our problem (which might explicitly relate arbitrary scores to statistical parameters) will lead to scores that yield better performance. Other changes in the dPUC scoring system may also lead to improvements; for example, differentiating between the two orderings of domain pairs is a promising avenue for future research, as it has been shown that many domain pairs occur in a single order [18].

While we applied dPUC to Pfam using HMMER2, there are other tools available which could be used, including SAM [45] and the new HMMER3 [21], as well as other domain databases, like SMART, Superfamily, and CDD. More broadly, the dPUC framework could be used to improve prediction of other protein sequence features, including signal sequences and transmembrane domains, both of which co-occur with a limited number of domains. The same underlying mathematical framework may also be applicable in other diverse settings, for example, in uncovering cis-regulatory modules, as transcription factors often work cooperatively to regulate genes. We have shown that dPUC significantly improves predictions over other models that score domains independently. We anticipate that our framework will be useful for incorporating context with other DNA, RNA, or protein sequence features and will find its greatest utility in the annotation of newly sequenced genomes from highly diverged organisms.

## Conclusions

Common domain identification methods typically consider each domain separately, and they have limited applicability when the similarity between the query sequence and known domains is very low, as is the case for poorly understood genomes. Here we significantly improved domain predictions by exploiting the tendency for domains to co-occur in specific combinations. We developed an approach based on the observation that weak domain predictions are better supported if they appear in previously-observed combinations, while domain combinations that have never been observed are less likely to be valid. Our method improved domain predictions in all organisms tested, including the best known model organisms. The biggest improvements were seen for the divergent organism *Plasmodium falciparum*, the parasitic agent of malaria, for which much of the core cellular machinery remains unidentified. Overall, our approach is likely to be most useful for poorly understood genomes where the need for additional annotations is arguably the greatest.

## Authors' contributions

AO and MS conceived and designed the approach and experiments. AO implemented the approach and performed the experiments. All authors analyzed the data, wrote, read and approved the final manuscript.

## Supplementary Material

Additional file 1 **Supplementary information**. PDF file (17 pages) that includes all the supplementary methods, results, figures and tables. Click here for file
