# Supplementary material for: Using context to improve protein domain identification
Source: BMC Bioinformatics. 2011 Mar 31;12:90. doi: 10.1186/1471-2105-12-90 (PMC3090354; doi:10.1186/1471-2105-12-90)
Supplement: Additional file 1 — Supplementary information. PDF file (17 pages) that includes all the supplementary methods, results, figures and tables. [file 1471-2105-12-90-S1.PDF]

Supplementary information

Using context to improve protein domain identification

Alejandro Ochoa, Manuel Llinás and Mona Singh

Supplementary methods

Pfam relevant details

The Pfam scoring system is complicated, so we present a summary of its features that are relevant for the work described here. Pfam is a database of HMMs representing protein and domain families. Each domain prediction has a "bit" score equivalent to the base-2 log-odds ratio of the maximum probability that the sequence was produced by the HMM versus the probability that it was generated by the background amino acid distribution. An *E*-value is calculated for each HMM based on its bit score distribution on random sequences, fit to an Extreme Value Distribution. Each family in Pfam version 23 can be predicted using a local (allowing fractional domains) or a glocal (forcing domains to be complete) HMM, and both local and global predictions come with manually curated thresholds on the bit scores called "gathering" thresholds. Lastly, each family and each mode (local and glocal) have two thresholds, one for each domain (represented here by T_i for domain i, with optional l or g superscripts when we want to focus on the local or glocal values), and one for the sum of all domains of each family (called "sequence" threshold in Pfam, represented here by T_F for family F, again with optional l or g superscripts).

Pfam Extended GA thresholds

The Standard Pfam GA thresholds produce a single datapoint with a fixed FDR and a fixed number of predictions. To estimate the performance of these non-context thresholds under different FDRs, we shift every threshold as follows. Choose a fixed "bit shift" d (we chose values between -10 and 10 in increments of 0.1). Then using

the same notation as before, every domain threshold T_i is now $T_i + d$, and similarly every sequence threshold T_F is now $T_F + d$. That way every d produces a new datapoint in our plots, with negative d producing more permissive thresholds with a higher FDR, and positive d producing more stringent thresholds with a lower FDR relative to the Standard Pfam. Predictions were made on the "local and glocal consolidated" domain set explained below.

dPUC implementation details

a. Local and glocal consolidation. We made Pfam predictions with HMMER2, $E \leq 1000$, on each proteome. Standard Pfam predictions were obtained using the curated gathering thresholds, and choosing between overlapping local and glocal predictions of the same family when both pass thresholds by using the curated ordering rules for each family (which might be to choose the one with the smallest E-value, or always favoring either the local or glocal prediction). For all domain predictions excluding the Standard Pfam, we consolidated the local and glocal predictions into a single set without applying thresholds, as follows. If a glocal domain prediction overlaps local domain predictions of the same family, we resolve overlaps as follows. Let the glocal score be denoted by H^g, its Pfam domain threshold be T^g, and the (potentially multiple) local scores be H_i^l with domain threshold T^l. We keep the glocal prediction if

$$H^g - T^g > \Sigma_i \, (H_i^l - T^l),$$

otherwise we keep the local predictions. This selects the predictions with the greatest total score ("normalized" by the thresholds). Very rarely, this procedure leads to domain loss, since it does not consider the Pfam "sequence" threshold when comparing scores, so a domain that passed both (domain and sequence) thresholds may be replaced by a domain that does not. However, when the sequence threshold is

the same as the domain threshold for both modes, this procedure is guaranteed to keep domains that pass both thresholds.

b. Positive elimination. The initial predictions may be too numerous for our direct approach to work efficiently, so they are pruned iteratively as follows. The full set of domains is P, but all domains that pass the Pfam gathering thresholds pass this filter automatically, and the rest of the domains are candidate domains. For every candidate domain i, let its score be H_i, and its context scores with the rest of the domains j be C_{ij}. Define the domain i's "total positive score", given P, as

$$S_{i,P}^{+} = H_i - T_i + \Sigma_{j \in P} \max\{0, C_{ij}\} \quad \forall i.$$

Note that this score is an upper bound of the score $S_{i,D}$ for every $D \subseteq P$, including the subset of domains D that optimizes the dPUC problem. We check if these positive scores satisfy the two Pfam thresholds,

$$S_{i,P}^{+} \geq 0 \qquad \forall i \text{ and}$$

$$\Sigma_{i \in F} S_{i,P}^{+} + T_i \geq T_F^{*} \quad \forall \text{ families } F,$$

where T_F^{*} is the average value of the local and glocal thresholds T_F^{l}, T_F^{g}, where this average is weighted by the number of each type of prediction for the domains in family F in the sequence. Domains that do not pass these thresholds in this optimistic scenario that ignores negative scores and allows overlaps, will not pass in the general problem, so they are eliminated from P. This elimination is iterated, reducing the problem by reducing $|P|$, until we converge to a set of domains P that do not allow further elimination. Each iteration runs in $O(|P|^2)$ time, and in practice the number of iterations is small even for $E \leq 1000$.

c. ILP. After our positive elimination, we solve the remaining hard combinatorial optimization problem using lp_solve 5.5.0.14 [1]. Note that T_F^{*} as defined above cannot be updated with the ILP problem, since this threshold is a non-linear function

of the x_i variables, and is instead set to the average value of the local and glocal thresholds for the domains in the family *F* present in the input, as a reasonable approximation.

In most cases, lp_solve can find the optimal solution to this maximization problem. However, these problems become too hard if too many domains are present, so we force lp_solve to timeout in 60s, and try a hierarchy of heuristics based on simplified problems as lp_solve continues to timeout. After the first timeout, we remove all "disallowed" overlaps, keeping the domains with the lowest *E*-values. After the second timeout, we remove all candidate domains, so only domains that pass the Pfam thresholds are left, but we run lp_solve so domains with negative context are eliminated. After the third timeout, we simply return the domains that pass Pfam thresholds without using context. We note that for *P. falciparum* with $E \leq 1$ all but one protein were solved using the original ILP, and PF11_0506 (containing 149 repeats of the Ag332 family) was solved after the first timeout.

nCODD context method

For the purpose of further comparison, in this work we introduce a novel double positive and negative filter, nCODD ("negative CODD"). Compared to CODD, here we additionally use negative context pairs, which are the complement of positive domain pairs limited to domains observed in the filtered architectures. We use the same notation of sets here as in the original CODD (see **Methods** in main text). First, for every domain *d* in *G* sorted ascending by *E*-value, *d* is eliminated if it has negative context with domains in *G* with lower *E*-values. We proceed by setting *P* = *G*. Lastly, for each domain *d* in *D* sorted ascending by *E*-value, we transfer *d* to *P* if *d* has positive context with any domain in *G*, and *d* does not have negative context or disallowed overlaps with any domain in *P*. Return *P* when done.

Estimated FDR details

This benchmark cannot be used for low-complexity proteins, as their shuffled sequences are similar to the original sequences. For this reason, the *P. falciparum* protein PF10_0374 had to be ignored in our benchmark; its extreme amino acid composition (38% E, 24% V, 10% P, 9% I, 7% L) led to shuffled sequences with high confidence predictions of the small Pfam repeat NPR (PF07391), which is also found in the real protein.

Supplementary results

Novel domain predictions may lead to GO term deletions with MultiPfam2GO

Our example, the *P. falciparum* protein PF11_0197 has two Ank repeats in the Standard Pfam, which lead to the prediction of the molecular function “binding” and biological process “cellular process”. Using dPUC adds an ACBP (Acyl CoA binding protein) domain, which refines the molecular function to “acyl-CoA binding”, but the biological process “cellular process” is deleted, since MultiPfam2GO did not find that proteins containing this architecture possess this GO term with a probability greater than their threshold. Reiterating, although Ank-containing proteins also contained the “cellular process” annotation with a high probability, proteins containing both Ank and ACBP enriched for proteins without this annotation, enough that the probability of the annotation drops under the predictive threshold. This is clearly an artifact resulting from incomplete GO annotations, and MultiPfam2GO responds adequately by changing the hypothesis and deleting the GO term. Moreover, “cellular process” is a very broad GO term, and deleting it results in very little loss of information.

dPUC Pfam parameter robustness

In the case of *P. falciparum*, we also made predictions with Pfam HMMs in which the null amino acid distribution matched the organism’s amino acid distribution (using hmmbuild --null, followed by hmmcalibrate). However, we saw a diminished

performance in both the version of those predictions keeping the "gathering" thresholds, or using *E*-value thresholds. Therefore, we did not consider that potential solution any further (data not shown).

There are many parameters controlling the scoring of our dPUC method (we only show the best set of parameters in **Figure 2 and Figure 3**). We could not exhaustively explore the large combinatorial space of parameters, but we tried many reasonable combinations. Surprisingly, we saw little sensitivity in performance when varying many of our parameters, including whether we removed single-instance architectures or not, and whether we used pair counts or "normalized" pair counts to estimate the pair distributions (data not shown). We saw larger fluctuations in the performance across organisms (sometimes the former method performed similar, and other times markedly worse than the latter) if we chose the marginal probabilities ($p_i = \Sigma_j p_{ij}$) as background instead of the uniform distribution ($p_i = 1/n$), or if we chose to count each architecture as a protein instead of counting every protein as a protein (data not shown). Lastly, we noticed that scaling the context scores (relative to the HMMER2 scores) by factors larger than 1 (up to 50) did not improve upon 1, while factors smaller than 0.5 worsened performance (data not shown).

We tried using other thresholds instead of the gathering thresholds in our context scoring model, for example bit thresholds equivalent to *E*-value thresholds (derived from the Extreme Value Distribution parameters that are pre-calculated in the Pfam database). However, the shape of the curve did not change significantly, but it was always shifted relative to its starting point (data not shown). Therefore, using the highest starting point, namely the gathering thresholds point, gives reasonable performance.

New annotations on *P. falciparum*

Using dPUC with $E \leq 1$, we found 515 new domain instances across the proteome of *P. falciparum*. While all novel domains contribute to increased amino acid coverage, not all of these refine functional predictions. In particular, domain family repeats are ignored by MultiPfam2GO [2], so only proteins with new "domain families" (as opposed to "domain instances") may have new functional annotations by such an approach. Therefore, we compiled the 196 *P. falciparum* proteins with 223 novel domain families (new Pfam family and clan), containing 317 new domain instances (including repeats). To assess the novelty of our predictions, we compared them to the current gene descriptions and domain predictions from Superfamily and SMART as included in PlasmoDB 6.0 [3]. We also used the OrthoMCL 4.0 database [4] to ask whether our *P. falciparum* functional predictions were coherent with Standard Pfam domains in orthologs. In the cases in which OrthoMCL and the conservation of our domain architectures predicted a single copy of a *P. falciparum* protein matched to one or more copies on other organisms, we predicted these proteins to be orthologous (protein pairs with a single ancestor separated by speciation; the multiple copies on other organisms are predicted to be in-paralogs, or recent duplications, by the criteria that OrthoMCL employs). We manually annotated all the proteins whose annotation could be improved from their PlasmoDB annotation, by narrowing down predictions with the combination of domain architectures and orthology predictions.

We reannotated 18 proteins using novel domains not predicted by any other domain database, but which have architecture and ortholog support (**Tables 3 and S3**). In these cases, only dPUC predicted crucial domains that complete the architecture and elucidate a specific function, which is often confirmed by our orthology analysis. Of note, nucleolar proteins, involved in ribosomal biogenesis, stood out from our predictions. To ensure that these predictions did not conflict with existing annotations,

we compiled all known nucleolar proteins in *Plasmodium* and their homologous yeast proteins (data not shown), and we confirmed that our predicted nucleolar proteins co-express with the known nucleolar proteins in the intraerythrocytic developmental cycle (data not shown).

Additionally, 38 proteins with descriptions of "unknown function" in PlasmoDB 6.0 had novel domains compared to Pfam, and these predictions were supported by Superfamily or SMART domains (data available on PlasmoDB and our website; **Table 4**). Combining our new domain architectures with ortholog predictions (**Table S3**), we were able to give descriptive names to these proteins, including homology to characterized proteins from other species, and to define specific molecular functions or biological processes when such functional predictions were sufficiently narrowed in our search.

The majority of our predictions fall in 124 proteins, which contained novel Pfam families whose presence was actually implied by the protein's current annotation and supported by other databases (Superfamily or SMART; data not shown), attesting to the high quality of our predictions. Fourteen family predictions were not supported by any of the evidence we considered, so they are likely false positives (data not shown). In most cases, these false predictions correspond to small repeats, they tend to fall in low-complexity regions, and some do not imply any functions. In other cases these predictions resemble known architectures, but these new domains are only a small portion of their HMMs (and the protein does not have enough space to fit the entire domain), suggesting spurious predictions.

In total, we predicted 223 new domain families, of which 209 are high confidence predictions (in most cases with multiple lines of evidence), while 14 are probably false positives. We therefore estimate that at most 6.3% of novel family predictions

for *P. falciparum* are incorrect. We expect the discrepancy between this and the estimated FDR for the new domain instances (expected to be 1.6%; **Table S1**) to be due to our inability to model low complexity regions in our shuffled protein sequences, which were very common among our false positives. We suggested improved annotations for 56 proteins, which we have contributed to the PlasmoDB website as "community annotations."

Supplementary references

1. **lp_solve: Open source (Mixed-Integer) Linear Programming system** [http://lpsolve.sourceforge.net/].

2. Forslund K, Sonnhammer ELL: **Predicting protein function from domain content**. *Bioinformatics* 2008, **24**:1681-1687.

3. Aurrecoechea C, Brestelli J, Brunk BP, Dommer J, Fischer S, Gajria B, Gao X, Gingle A, Grant G, Harb OS, Heiges M, Innamorato F, Iodice J, Kissinger JC, Kraemer E, Li W, Miller JA, Nayak V, Pennington C, Pinney DF, Roos DS, Ross C, Stoeckert CJ, Treatman C, Wang H: **PlasmoDB: a functional genomic database for malaria parasites**. *Nucl Acids Res* 2009, **37**:D539-543.

4. Chen F, Mackey AJ, Jr CJS, Roos DS: **OrthoMCL-DB: querying a comprehensive multi-species collection of ortholog groups**. *Nucleic Acids Res.* 2006, **34**:D363–D368.

Supplementary figures

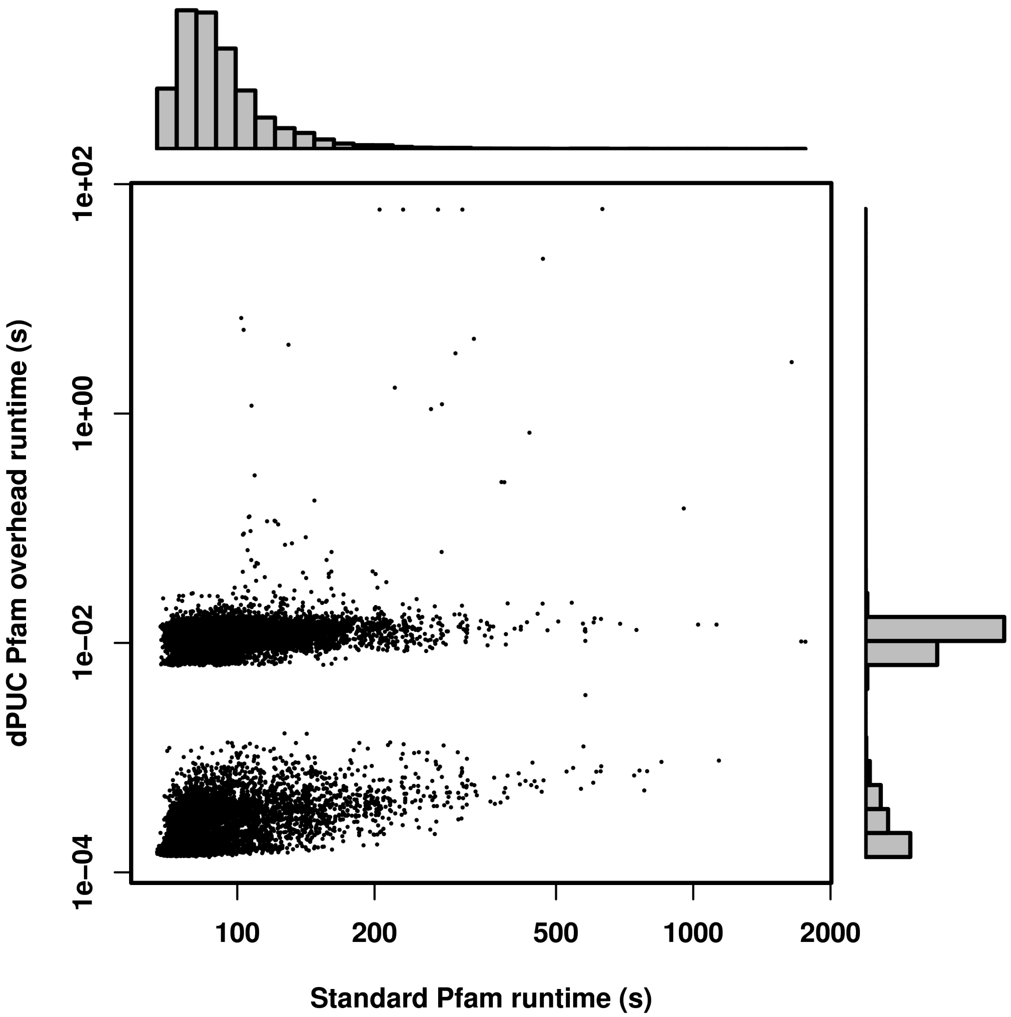

Figure S1 - Standard Pfam and dPUC Pfam runtime analysis

Each point corresponds to one of the 25,047 proteins of the combined proteomes of *E. coli*, *M. tuberculosis*, *S. cerevisiae*, and *P. falciparum*. Computations were performed on a 2.66 GHz Intel processor with 8GB RAM. Runtime is measured in wall clock time. The Standard Pfam runtime (x-axis) includes HMMER runtime, which takes the bulk of the time, as well as the Standard Pfam processing (removing the domains that do not pass the Pfam gathering thresholds, removing overlaps between local and glocal predictions according to Pfam family-specific rules, and removing overlaps between domains of the same Pfam clan by keeping the domains with the lowest *E*-

value). The dPUC Pfam overhead runtime (y-axis) excludes HMMER runtime, in order to show the small effect of incorporating dPUC relative to standard domain prediction. Both axes are show in log scale to accommodate the long tails of the distributions. Note that the dPUC runtime is bimodal: problems that require lp_solve are clustered around 0.01 s, while problems that do not require lp_solve (when the positive elimination removes all domain predictions, see **Methods**) are clustered around 0.0001 s.

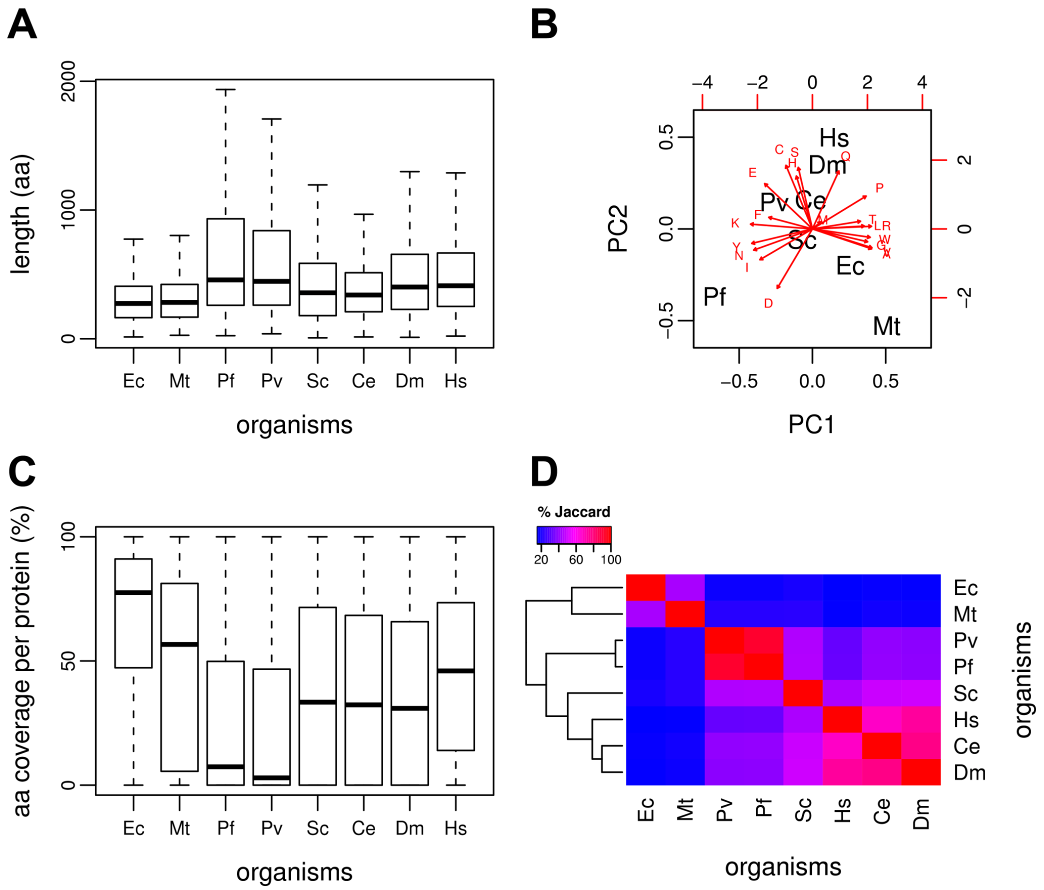

Figure S2 - Comparison of global properties of test organisms

A. *Plasmodium* sp. have longer proteins, bacteria have smaller ones. In these boxplots of protein length distributions, the thick bar represents the median, the bottom and top of the box are the lower and upper quartiles, the plot whiskers extend to the most extreme datapoint which is no more than 1.5 times the interquartile range

from each box, and the outliers are not plotted. **B. *P. falciparum*, *M. tuberculosis*, and *H. sapiens* have extreme amino acid compositions, *S. cerevisiae* has an average composition.** This Principal Component Analysis plot of the average amino acid compositions per organism was generated using the functions prcomp and biplot from the R statistics package. The two largest principal components are plotted for each organism (black letters), and the amino acid axes are projected onto these two principal components (red letters and arrows). **C. *E. coli* proteins are well-covered by domains, *Plasmodium* proteins are the least covered.** Coverage taken from Standard Pfam domain predictions. This pattern may explain why dPUC Pfam predicts the most new domains in *Plasmodium* species and the least in *E. coli*: there are more domains left to be discovered in *Plasmodium* species, while *E. coli* leaves little room for novel discoveries. Meaning of bars, box, whiskers, and circles is the same as in panel A. **D. Bacteria and Eukaryotes share only ~20% of Pfam families.** Similarity of Pfam family content is measured by the *Jaccard Index = I/U*, in percents, where *I* is the list of Pfam families common to both organisms, and *U* is the union of Pfam families of both organisms. Image produced with heatmap.2 from the R statistics package, clustered using hierarchical clustering over Euclidean distance.

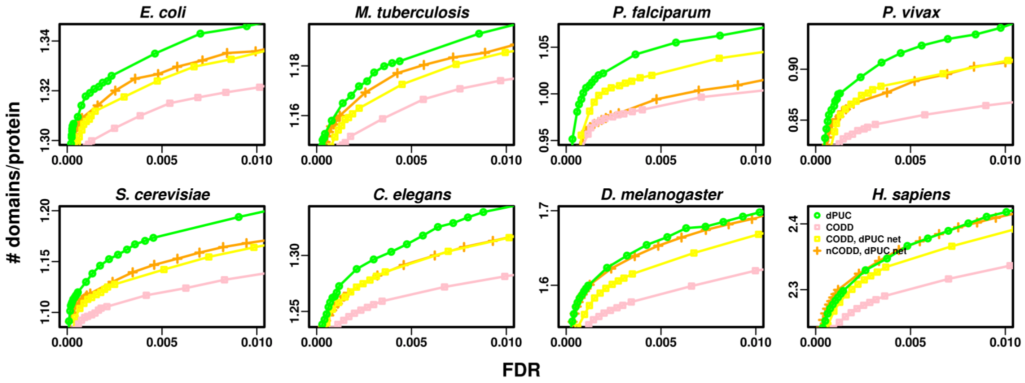

Figure S3 - FDR analysis of dPUC and CODD variations to determine features important for performance

dPUC and CODD are as in **Figure 2**. In addition, we ran CODD using the more complete dPUC positive context network instead of the original CODD network (yellow squares), improving performance. Lastly, we introduced nCODD as a modification of CODD that exploits negative context, also ran with the dPUC network (orange "+").

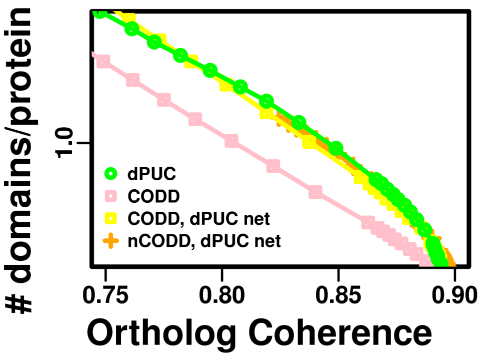

Figure S4 - Ortholog Coherence analysis of dPUC and CODD variations to determine features important for performance

All methods and colors are the same as in **Figure S3**, and the Ortholog Coherence test is the same as in **Figure 3**.

Supplementary tables

Table S1 - Coverage of predictions by Standard Pfam and dPUC Pfam

	E. c.	*M. t.*	*P. f.*	*P. v.*	*S. c.*	*C. e.*	*D. m.*	*H. s.*
**Total**								
Proteins	4,362	7,945	5,396	5,396	7,343	23,073	16,415	20,318
Amino acids	1,373,030	2,660,223	4,094,366	3,750,741	3,289,690	10,018,548	8,860,453	11,244,964
**Standard Pfam**								
Domains	5,465	8,640	5,000	4,242	7,742	27,170	24,073	43,575
Doms. no reps	5,081	7,913	3,737	3,463	6,666	19,806	15,925	25,356
Doms. reps	384	727	1,263	779	1,076	7,364	8,148	18,219
Amino acids	906,388	1,366,966	745,934	637,697	1,266,350	3,740,085	2,985,558	4,519,325
	(66.01%)	(51.39%)	(18.22%)	(17.00%)	(38.49%)	(37.33%)	(33.70%)	(40.19%)
Proteins	3,809	5,977	2,947	2,748	4,971	15,031	11,455	16,534
	(87.32%)	(75.23%)	(54.61%)	(50.93%)	(67.70%)	(65.15%)	(69.78%)	(81.38%)
FDR	0.000101	0.000191	0.000860	0.000695	0.000226	0.000248	0.000287	0.000319
**dPUC Pfam**								
Domains	5,700	9,158	5,515	4,728	8,223	29,363	26,258	46,692
Doms. no reps	5,214	8,143	3,951	3,657	6,891	20,530	16,622	26,225
Doms. reps	486	1,015	1,564	1,071	1,332	8,833	9,636	20,467
Amino acids	927,991	1,423,372	800,037	686,553	1,306,095	3,917,358	3,153,737	4,683,468
	(67.59%)	(53.51%)	(19.54%)	(18.30%)	(39.70%)	(39.10%)	(35.59%)	(41.65%)
Proteins	3,815	5,982	3,000	2,784	4,990	15,136	11,523	16,626
	(87.46%)	(75.29%)	(55.60%)	(51.59%)	(67.96%)	(65.60%)	(70.20%)	(81.83%)
FDR	0.000333	0.000437	0.001949	0.001280	0.000565	0.001182	0.001160	0.001445
FDR, new doms only	0.008079	0.007731	0.016254	0.008393	0.008955	0.013861	0.012632	0.020171

Domains No Repeats counts each family only once per protein, while Domains Repeats counts only extra instances of domains ignoring the first appearance in each protein.

Table S2 - Comparison of Gene Ontology predictions between Standard Pfam and dPUC

	E. c.	*M. t.*	*P. f.*	*P. v.*	*S. c.*	*C. e.*	*D. m.*	*H. s.*
**GO terms**								
Same	10,840	16,413	7,581	7,172	13,841	36,282	30,817	47,424
New	134	233	303	297	191	856	809	1,017
More specific (standard)	46	76	59	59	65	217	241	300
More specific (context)	45	76	61	64	77	224	253	321
Less specific (standard)	18	40	12	24	22	137	220	196
Less specific (context)	16	40	11	24	24	135	211	213
Deleted	34	70	65	104	60	324	274	343
**GO terms (%)**								
Same	99.1	98.88	98.24	97.46	98.95	98.17	97.67	98.26
New	1.23	1.40	3.93	4.04	1.37	2.32	2.56	2.11
More specific (context)	0.41	0.46	0.79	0.87	0.55	0.61	0.80	0.67
Less specific (standard)	0.16	0.24	0.16	0.33	0.16	0.37	0.70	0.41
Deleted	0.31	0.42	0.84	1.41	0.43	0.88	0.87	0.71
**Proteins**								
Same	3,560	5,668	2,530	2,380	4,627	12,476	10,576	15,499
New	35	72	101	94	64	286	263	325
More specific	12	12	10	10	11	60	40	78
New and more specific	18	30	21	20	21	49	71	98
Less specific	4	14	3	2	6	36	54	52
Deleted	7	14	19	24	9	62	50	71
Deleted and less specific	3	2	1	5	0	18	19	15
Mixed	10	28	28	38	34	151	177	218

When counting GO terms, each category is mutually exclusive except for “more specific standard/context” and “less specific standard/context”. Each GO term in “more specific (standard)” (from the Standard Pfam) can be matched with at least one more specific GO term in “more specific (context)” (from dPUC), but these two counts do not match because becoming more specific is not always a one-to-one relationship. Similarly, for “less specific (standard)” and “less specific (context)” (one GO term can be mapped to multiple less specific terms). All GO term percents are relative to the number of GO terms in the Standard Pfam for each organism, and each “percent” category is mutually exclusive. When counting proteins, all categories are mutually exclusive. “Mixed” means that both “new or more specific” and “deleted or less specific” GO terms occurred in the same protein.

Table S3 - Novel dPUC Pfam predictions agree within orthologous groups

Protein ID	Suggested reannotation (this study, duplicated from Tables S3 and S4)	OrthoMCL 4.0 groups (curated in parentheses if different)	Phylogenetic range (OrthoMCL 4.0, and curated in parenthesis if different)
PFL0980w	Debranching enzyme-associated ribonuclease (DRN1 ortholog), putative	OG4_12978	Eukaryota, except Euglenozoa
PF13_0222	RNA lariat debranching enzyme (DBR1 ortholog), putative	OG4_11656	Eukaryota
PF11_0086	Poly(A)-binding protein-interacting protein 1 (PAIP1 ortholog), putative	OG4_11313 (OG4_11313 Apicomplexa, OG4_18259 Metazoa)	Apicomplexa, Fungi (Apicomplexa, Metazoa)
PFE1390w	Post-translational mRNA regulation (ABSTRAKT ortholog), putative	OG4_13067	Eukaryota, except Fungi
PF08_0130	U3 ribonucleoprotein component (PWP2 ortholog), putative	OG4_11482	Eukaryota
PF14_0456	U3 ribonucleoprotein component (DIP2 ortholog), putative	OG4_11396	Eukaryota
PF10_0128	U3 ribonucleoprotein component (UTP13 ortholog), putative	OG4_11588	Eukaryota
PFI1025w	U4/U6 snRNA-associated-splicing factor (PRP24 ortholog), putative	OG4_34515 (OG4_34515 Apicomplexa, OG4_13068 Eukaryota, except Apicomplexa and Euglenozoa)	Apicomplexa (Eukaryota, except Euglenozoa)
PFL0985c	Ribosome biogenesis regulator (TSR3 ortholog), putative	OG4_11113	Eukaryota and Archaea
MAL8P1.19	Ribosomal biogenesis RNA helicase protein (DBP10 ortholog), putative	OG4_11705	Eukaryota
PFE0560c	Atypical Golgi transport protein (AVL9 ortholog) with MORN domains, putative	OG4_42850 (OG4_42850 *Plasmodium*, OG4_50280 Piroplasmida, OG4_114378 Sarcocystidae, OG4_13875 Metazoa, Fungi, other Eukaryota)	*Plasmodium* (Alveolata, Fungi, Metazoa, other Eukaryota)
PFL1455w	Vacuolar transporter chaperone (VTC2/3/4 ortholog), putative	OG4_14575 (OG4_14575 Apicomplexa, OG4_14447 Eukaryota, except Metazoa, OG4_21484 and OG4_52804 additional Fungi)	Eukaryota, except Metazoa
PFL2255w	DNA replication origin binding protein (DIA2 ortholog), putative	OG4_55945 (OG4_55945 Apicomplexa, OG4_46325 Fungi, maybe OG4_13521 Metazoa, Fungi, Viridiplantae)	Apicomplexa (Apicomplexa, Fungi)
PFF1070c	Ribosome or tRNA methylthiotransferase (RIMO or MIAB ortholog) or CDK5 regulatory subunit-associated protein 1, putative	OG4_10254	Bacteria, Archaea, and Eukaryota, except Fungi
PFL1045w	FbpA domain protein, putative	OG4_34378 (maybe related to OG4_11062, Eukaryota and Archaea)	Aconoidasida (may be related to an Eukaryota and Archaea group)
MAL13P1.182	GID8 ortholog, putative	OG4_11912	Eukaryota, except Euglenozoa
MAL13P1.79	CCCH zinc finger protein, putative	OG4_23238	Apicomplexa
MAL13P1.37	Tripartite motif protein, putative	OG4_37704	Aconoidasida
PFE1240w	Wybutosine synthesis protein (TYW1 ortholog), putative	OG4_11477	Eukaryota and Archaea
PFF1490w	Tetrahydrofolate dehydrogenase/cyclohydrolase (MTD1 ortholog, MIS1/ADE3 homolog without FTHFS domain), putative	OG4_47225 (OG4_47225 *Plasmodium*, OG4_114990 Sarcocystidae, OG4_18660 Fungi, OG4_51093 and OG4_118871 Viridiplantae, maybe OG4_10140 Bacteria, Eukaryota except Apicomplexa, some Archaea)	*Plasmodium* (Eukaryota and Bacteria, some Archaea)
MAL8P1.139	Regulator of (H+)-ATPase in Vacuolar membrane (RAV1 ortholog), putative	OG4_24797 (OG4_24797 *Plasmodium*, OG4_20682 Fungi, OG4_14510 Metazoa)	*Plasmodium* (Eukaryota)
PF08_0124	CACTIN homolog, putative	OG4_12820	Eukaryota, except Euglenozoa
PF10_0152	Non-canonical cytoplasmic specific poly(A) RNA polymerase protein (CID13 ortholog), putative	OG4_12495	Eukaryota, except Euglenozoa
MAL13P1.170	Non-canonical poly(A) RNA polymerase protein (PAP2/TRF5 ortholog), putative	OG4_10880	Eukaryota
PFI1560c	Required for mitochondrial morphology (MAM3 ortholog), putative	OG4_10104	Eukaryota and Bacteria, some Archaea
PF10_0126	Phosphoinositide binding protein (HSV2/ATG18 ortholog), putative	OG4_11612	Eukaryota, except Euglenozoa
PFI0510c	DNA repair protein (REV1 ortholog), putative	OG4_80162 (OG4_80162 *Plasmodium*, OG4_12179 Eukaryota except most Apicomplexa)	*Plasmodium* (Eukaryota)
MAL13P1.54	Alternative splicing regulator (SMU-1 ortholog), putative	OG4_12877	Eukaryota, except Euglenozoa and Fungi
PF14_0052	COBW domain-containing protein 1 (CBWD1 ortholog), putative	OG4_10840	Eukaryota and Bacteria, some Archaea
PF08_0012	Histone lysine N-methyltransferase,	OG4_33853 (OG4_33853 Apicomplexa,	Apicomplexa (Apicomplexa

	putative	OG4_33231, OG4_36931, OG4_50963, OG4_51096, OG4_90967, and OG4_100563 Viridiplantae)	and Viridiplantae)
PFE1445c	T-cell immunomodulatory protein (human TIP homolog), putative	OG4_13053	Metazoa and Apicomplexa, some Fungi and other Eukaryota
PFL0975w	Unconventional myosin fused to IQ and RCC1 domains, putative	OG4_28078 (OG4_28078 *Plasmodium* and *Tetrahymena*, OG4_10145 Apicomplexa only)	*Plasmodium* and *Tetrahymena* (Alveolata)
PF11_0276	Steryl ester hydrolase (TGL1/YEH1/YEH2 ortholog), putative	OG4_36130 (OG4_36130 *Plasmodium*, OG4_10339 Metazoa, Fungi, Viridiplantae, OG4_22233 additional Fungi, and very many more small groups for Metazoa)	*Plasmodium* (Eukaryota, except Euglenozoa)
PF13_0190	Chaperone binding protein, putative	OG4_22006	Apicomplexa
PF11_0287	CRAL/TRIO protein, putative	OG4_19268	Apicomplexa
PF11_0197	Acyl-CoA-binding protein, putative	OG4_13447	Eukaryota, except Euglenozoa
PF14_0647	Rab GTPase activator, putative	OG4_23919 (OG4_23919 Apicomplexa, OG4_16111 Metazoa, OG4_64715 *Trichomonas*)	Apicomplexa (Apicomplexa and Metazoa)
PFL0575w	PHD finger and flavin containing amine oxidoreductase, putative	OG4_25486	Apicomplexa
MAL13P1.246	E1-E2 ATPase, putative	OG4_42315	*Plasmodium*
PF11_0116	Nol1/Nop2/Fmu-like protein, putative	OG4_12812	Metazoa, Apicomplexa, and Euglenozoa
MAL7P1.127	Rab GTPase activator and protein kinase, putative	OG4_30676 (OG4_30676 Apicomplexa, OG4_15147 Metazoa and a few more Apicomplexa)	Apicomplexa (Apicomplexa and Metazoa)
PFC0425w	PHD finger protein, putative	OG4_27967	*Plasmodium*
PFI0975c	Regulator of chromosome condensation, putative	OG4_47073	*Plasmodium*
PFD0900w	Regulator of chromosome condensation, putative	OG4_48398	*Plasmodium*
MAL7P1.132	Protein kinase, putative	OG4_42790	*Plasmodium*
PFF0810c	Ras GTPase, putative	OG4_48492	*Plasmodium*
PFL1990c	RNA binding protein, putative	OG4_33248	*Plasmodium*
PF07_0066	RNA binding protein, putative	OG4_20129	Alveolata, other Eukaryota
PF13_0147	RNA binding protein, putative	OG4_20130	Alveolata
PFF1120c	EGF-like membrane protein, putative	OG4_21327	Apicomplexa, some Metazoa
PF14_0262	WD40 and TPR repeats protein, putative	OG4_14359	Eukaryota, except Euglenozoa
PFI0275w	WD40 repeat and EF hand protein, putative	OG4_33245	*Plasmodium*
PF10_0285	WD40 repeat protein, putative	OG4_48272	*Plasmodium*
PF11_0195	WD40 repeat protein, putative	OG4_43534	*Plasmodium*
PF14_0640	WD40 repeat protein, putative	OG4_12936	Eukaryota, except Euglenozoa
MAL13P1.308	ARM repeat protein, putative	OG4_22396	Apicomplexa

In many instances, OrthoMCL seems to partition true orthologous groups, so we joined them back as follows: the Pfam architectures on their sequences strongly agree within and across the selected groups, to the exclusion of all or most other orthologous groups, and the organisms present in these groups must not overlap (which is consistent with a partition).
